# Identification of *PGRMC1* as a Candidate Oncogene for Head and Neck Cancers and Its Involvement in Metabolic Activities

**DOI:** 10.3389/fbioe.2019.00438

**Published:** 2020-01-08

**Authors:** Yue Zhao, Xiangyan Ruan

**Affiliations:** Department of Gynecological Endocrinology, Beijing Obstetrics and Gynecology Hospital, Capital Medical University, Beijing, China

**Keywords:** *PGRMC1*, head and neck cancer, TCGA, oncogene, metabolic

## Abstract

Progesterone Receptor Membrane Component 1 (*PGRMC1*/Sigma-2 receptor) is located on chromosome Xq21 and encodes a haem-containing protein that interacts with epidermal growth factor receptor (EGFR) and cytochromes P450, with function in tumor proliferation and chemoresistance. Although the over-expression of *PGRMC1* reported in many different types of human cancers, systematic analysis of its oncogenic role of *PGRMC1* has not been performed for any cancer. In this work, we analyzed the transcriptomics, genomics, and clinical data of 498 head-neck squamous cell carcinoma (HNSC) samples from the public-accessible database, The Cancer Genome Atlas (TCGA). The Cox regression was performed to calculate the hazard ratio (HR) of *PGRMC1* expression as a prognosis feature for overall survival (OS). Our results demonstrated that *PGRMC1* expression served as a predictor for worse OS (HR = 1.95, *P* = 0.0005) in head-neck squamous cell carcinoma. And the over-expression of *PGRMC1* was strongly correlated with various metabolic process activity as well as cancer metastasis and cell proliferation features in human head-neck squamous cell carcinoma patient's cohort. Besides, the over-expression and unfavorable prognosis value of *PGRMC1* were also observed in many other cancer types. This study provides insights into the potential oncogenic functional significance of *PGRMC1* in cancer research.

## Introduction

*PGRMC1* belongs to the membrane-associated progesterone receptor (MAPR) gene family with a cytochrome b5-like heme/steroid-binding domain and encodes a putative membrane-associated progesterone steroid receptor protein. The protein anchored to the cell membrane through the N-terminal transmembrane helix and interacts with epidermal growth factor receptor (EGFR) and cytochromes P450 (Zheng et al., 2017). Many reports have implicated that the *PGRMC1* expressed predominantly in human liver, ovary, and kidney tissue, and is known to be over-expressed in various types of cancers, including breast cancer, head and neck cancer, ovarian cancer, and lung cancer (Kabe et al., 2016, 2018; Kim et al., 2019). Previous studies revealed that *PGRMC1* played important roles in cancer proliferation and regulation of the cancer cell susceptibility to chemotherapy (Kabe et al., 2016; Zhang et al., 2016; He et al., 2018). As a strong correlation between *PGRMC1* expression and tumor progression has been confirmed, it has become a novel and promising target of the therapeutic intervention for cancer treatments.

Head and neck squamous cell carcinoma (HNSC) is one of the most common types of human cancer, with an annual incidence of more than 500,000 cases worldwide (Leemans et al., 2018; Alsahafi et al., 2019). Despite surgery, radiation, and chemotherapy, approximately half of all patients will die of the disease (Bratman et al., 2016). HNSC is classified by its location: oral cavity, oropharynx, nasal cavity, and paranasal sinuses, nasopharynx, larynx, or hypopharynx. It develops through the accumulation of multiple genetic and epigenetic alterations in a multi-step process. The association of smoking and HNSC was observed in developing countries, while the human papillomavirus (HPV) as a critical role affecting non-smokers in the rise of HNSCC is emerging in developed countries (Begum and Westra, 2008; Desrichard et al., 2018). Recent studies revealed that the expression of *PGRMC1* dramatically elevated in head and neck cancer (Hampton et al., 2015). However, to our knowledge, the oncogenic role of the *PGRMC1* gene in head and neck cancer has not been systematically analyzed, and additional researches are merited.

The largest public-accessible cancer genomics database namely The Cancer Genome Atlas (TCGA) profiled nearly 500 head and neck squamous cell carcinoma samples to provide a comprehensive landscape of genomic alterations and clinical annotations. In order to gain a better understanding of the roles of the *PRGMC1* gene in head and neck cancer carcinogenesis, we explored expression profiling, copy number variation, somatic mutations, and clinic pathologic association significance of the *PRGMC1* gene from 498 HNSC samples obtained from the TCGA database. Our analysis suggests that *PGRMC1* seems a novel oncogene for HNSC since its correlation with signature genes of tumor proliferation, metabolism and tumor metastasis, and its over-expression could be used as an indicator for clinical unfavorable prognosis of HNSC patients and many other cancer types.

## Materials and Methods

### Datasets

**Figure 1:**
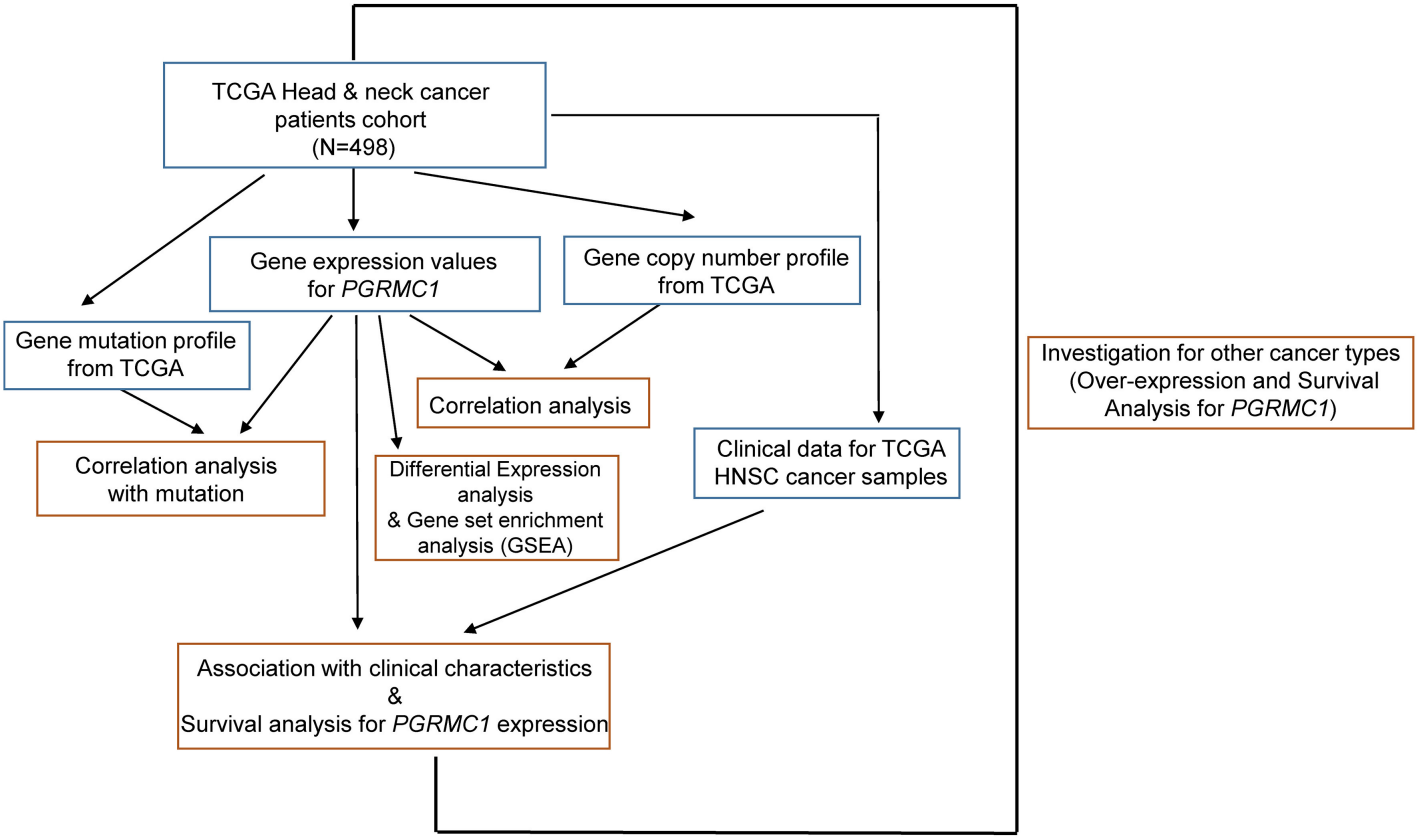
Illustration of study workflow. The flowchart of data collection and method implementation in this work.

The transcriptomics and genomic data, and detailed clinical information of HNSC cohort were based on the TCGA HNSC dataset (Figure 1). We accessed the TCGA data portal (https://portal.gdc.cancer.gov/, March 2019) and downloaded mRNA expression quantification profiles (HTSeq–FPKM) and masked copy number segment profiles for head and neck cancer (*N* = 498). GISTIC2 (Mermel et al., 2011) method was applied to the transformed copy number segment data, with a noise threshold used to determine copy gain or loss. The copy number values were obtained by examining the distribution of log_2_ ratios to identify peaks associated with copy number states. Clinical data files and annotated mutation files of cancer samples were downloaded from cBioPortal for Cancer Genomics (http://www.cbioportal.org/index.do, March 2019).

### Gene-Set Enrichment Analysis

Gene-set enrichment analysis was performed with the GSEA program (v. 3.0) (Subramanian et al., 2005). The Broad Molecular Signatures Database (MSigDB v6.0) set H (hallmark gene sets, 50 gene sets) and set CP:KEGG (KEGG gene sets, 186 gene sets) were used, which summarize and represent specific well-defined biological states and pathway processes. The GSEA program was run with 1,000 permutations for statistical significance estimation, and the default signal-to-noise metric between the two phenotypes was used to rank all genes.

### Survival Analysis

The Kaplan-Meier analysis for overall survival was proceeded based on the gene's expression level whose cut-off level was set at the quantile value (top 25% and bottom 25%) with the aid of GraphPad Prism 8 software and the Log-Rank was utilized to test. Univariate survival analysis was performed by using the Cox proportional hazard regression model with overall survival time (5-year) to assess the prognostic value (Supplementary Table 1). The prognostic value of discrete variables was estimated by Kaplan–Meier survival curves, and the log-rank test was employed to estimate the significance among different survival curves.

### Biostatistical Analysis

Data were analyzed by using an unpaired Mann-Whitney test to compare between two groups and one-way analysis of variance (ANOVA) for multiple comparisons. Fisher's exact test was used for enrichment analysis. The Pearson correlation coefficient was performed to estimate the strength and significance of the association between two continuous variables. DESeq2 (Love et al., 2014) framework was employed to perform the differential expression analysis between *PGRMC1*-high and *PGRMC1*-low HNSC samples. The multiple testing adjustments (False Discovery Rate, FDR < 0.05), log2 ratio of gene expression fold change (log2 Fold Change > 1) and the difference of the mean value of normalized counts for each gene (difference > 500) determined the significant difference. In the graphs, y-axis error bars represent median with 95% CI as indicated. Statistical calculations were performed using GraphPad Prism software (GraphPad Software, San Diego, California) or R software (https://www.r-project.org/).

### Ingenuity Pathway Analysis

The up-regulated genes with differential expression significance information in *PGRMC1*-high HNSC samples were used as input for the Ingenuity Pathway Analysis (IPA) software (Qiagen, Redwood City, CA). Then the canonical pathways and signal network analysis modules were performed by default parameters.

## Results

### Oncogenic Role and Prognosis Value of *PGRMC1* Over-expression

**Figure 2:**
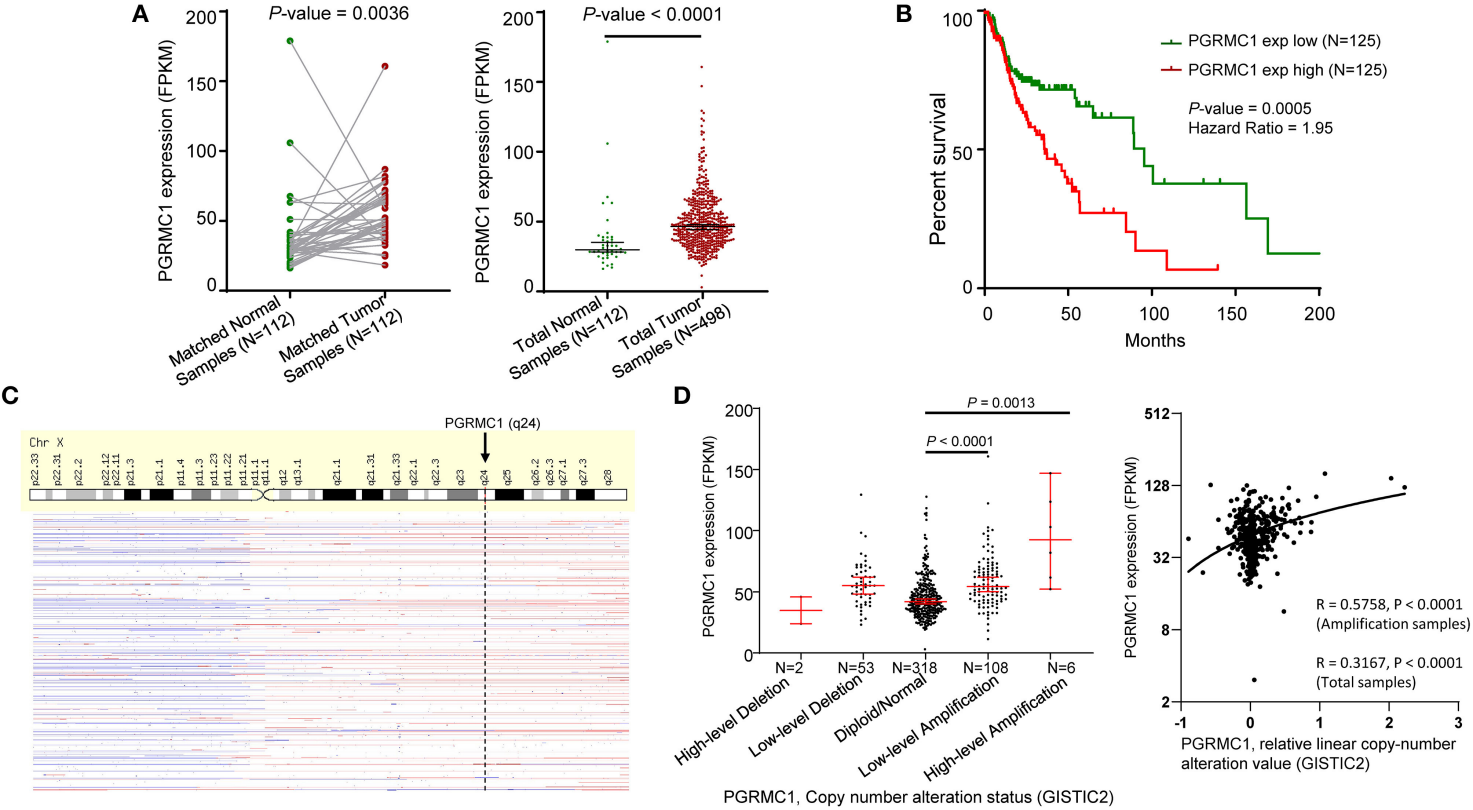
Oncogenic role of *PGRMC1* in human head and neck cancer. **(A)** Change of *PGRMC1* mRNA expression between tumor samples and normal samples from TCGA HNSC studies. **(B)** Kaplan–Meier survival curve comparing the high (*N* = 125) and low (*N* = 125) expression value of *PGRMC1* (determined by the quantile value) for the TCGA HNSC patient cohort. **(C)** GISTIC heat map showing genomic copy-number profiles from TCGA HNSC studies. Gain (red) and loss (blue) of each peak are shown. **(D)** Dot plot and correlation figure showing the positive correlation between *PGRMC1* copy number values defined by GISTIC2 approach and mRNA expression values quantified by FPKM (*N* = 498).

We first determined the mRNA expression of *PGRMC1* in head and neck cancer samples from the TCGA database and found that the transcripts of *PGRMC1* were significantly higher expressed in paired HNSC samples (*N* = 112) compared with adjacent normal tissues (*P* = 0.0035) (Figure 2A, left). The trend of upregulation was also observed when compared between unpaired tumor samples and all normal samples (*P* < 0.0001) (Figure 2A, right). Since *PGRMC1* is aberrantly up-regulated expressed in tumor samples, we next investigated the clinical implication of *PGRMC1* expression in HNSC patients. The distribution of *PGRMC1* expression in HNSC cohort was normal approximation (Supplementary Figure 1A), based on the Kaplan–Meier survival curves, as shown in Figure 2B and Supplementary Figure 1B, our data revealed that higher *PGRMC1* expression value (determined by quartile and median, respectively) significantly associated with worse overall survival in patients of the HNSC cohort. Moreover, considering the gene copy number amplification usually acted as a major genetic mechanism to enhance the expression of oncogenes, we interrogated the chromosomal segment value of head and neck cancer samples of the TCGA HNSC dataset to determine the significant copy number alterations. As expected, we observed the chromosomal region of Xq24 (encompassing *PGRMC1*) harbored abundant amplification events (Figure 2C). Then we stratified HNSC patients into five groups based on *PGRMC1* copy number values by using GISTIC2 framework (High-level Deletion, *N* = 2; Low-level Deletion, *N* = 53; Diploid, *N* = 318; Low-level Amplification, *N* = 108; High-level Amplification, *N* = 6). As shown in Figure 2D, more than one-fifth of all HNSC samples harbored *PGRMC1* amplification, and consistently, HNSC samples harboring *PGRMC1* amplification exhibited higher mRNA expression than those that exhibit diploid *PGRMC1*. Therefore, the gain of gene copy numbers likely to be one of the main machinery that makes a contribution to the over-regulation of *PGRMC1* in HNSC patients. In summary, a positive correlation between *PGRMC1* copy number amplification and mRNA over-expression was found among HNSC samples, and its over-expression is an adverse prognostic factor for HNSC patients. However, the significance was not reached when we compared the survival curves based on different copy number variation status (Supplementary Figure 1C).

**Figure 3:**
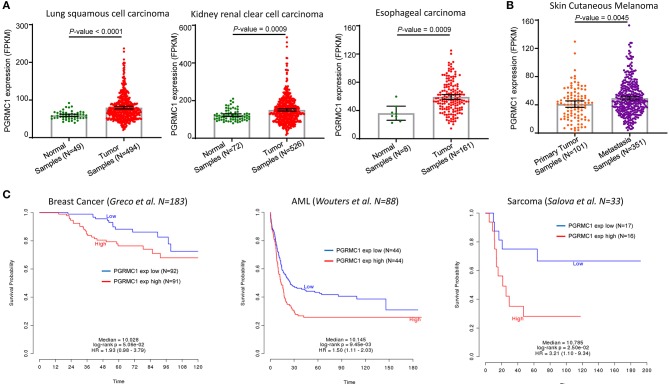
Prognosis value of *PGRMC1* in various cancer types. **(A)** Change of *PGRMC1* mRNA expression between tumor samples and normal samples from lung squamous cell carcinoma, kidney renal clear cell carcinoma, and esophageal carcinoma studies based on the TCGA database. **(B)** Change of *PGRMC1* mRNA expression between metastasis tumor samples and primary tumor samples from TCGA skin cutaneous melanoma studies. **(C)** Kaplan–Meier survival curve comparing the OS time for different *PGRMC1* expression subtypes in breast cancer, acute myelocytic leukemia, and sarcoma. Statistical significance was determined by the log-rank test.

Furthermore, to determine the general significance of the oncogenic and prognosis role of *PGRMC1*, we investigated the over-expression and prognosis value in many different cancer types. As shown in Figure 3A, based on the TCGA database, the expression value of *PGRMC1* in lung squamous cell carcinoma, kidney renal clear cell carcinoma, esophageal carcinoma was dramatically higher than matched normal samples, and the expression value in metastasis samples of skin cutaneous melanoma is significantly higher than primary tumor samples (Figure 3B). Moreover, a strong association between *PGRMC1* over-expression and worse overall survival of breast cancer (Muranen et al., 2011), acute myelocytic leukemia (Wouters et al., 2009), and sarcoma patients (Savola et al., 2011) was also observed (Figure 3C).

### *PGRMC1* Over-expression Is Associated With the Tumor Metabolism Activity

**Figure 4:**
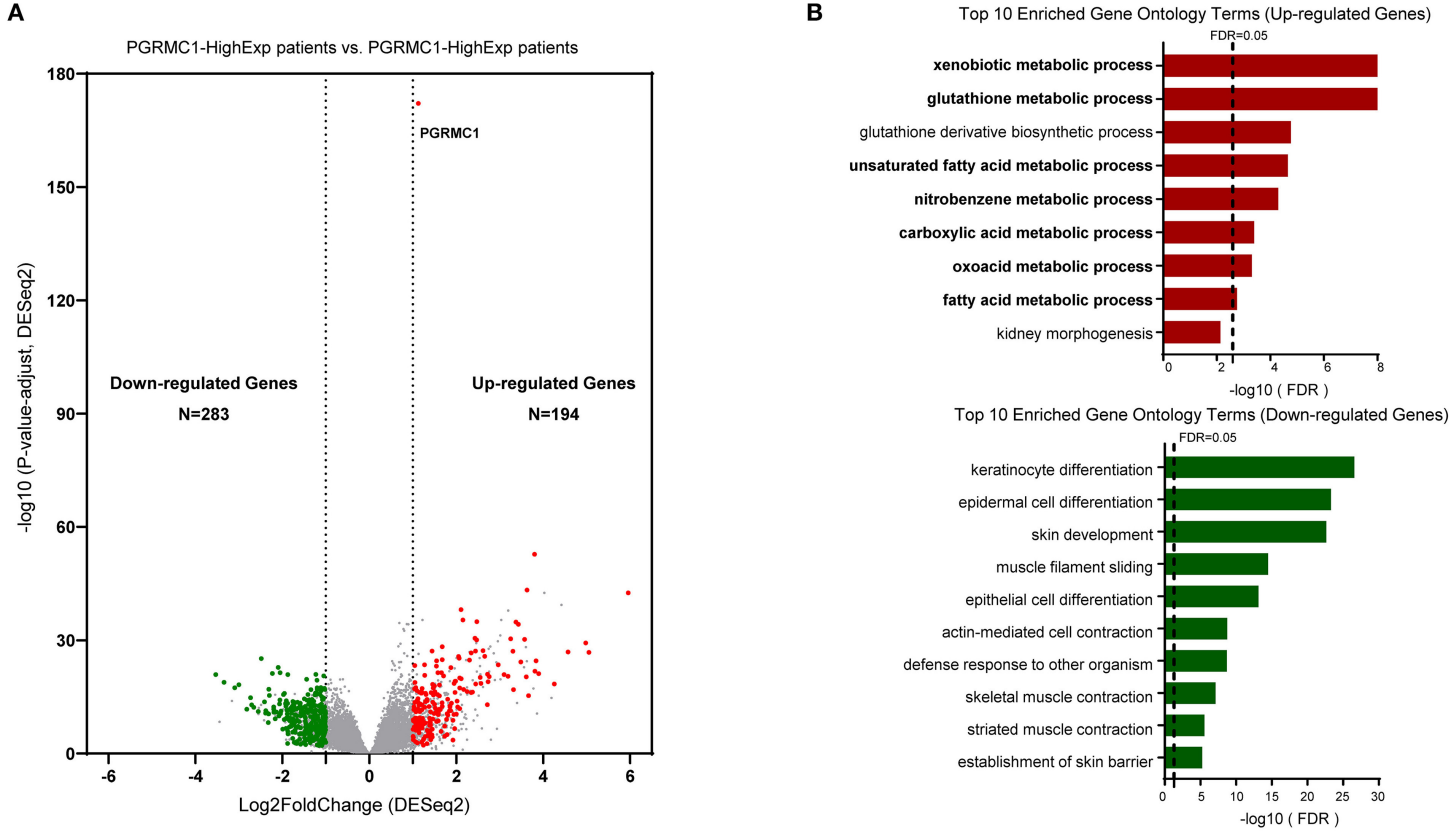
Identification of differentially upregulated expressed genes. **(A)** Volcano plot of mRNA expression changes between HNSC samples harboring *PGRMC1* high- and low- expression value. The x-axis specifies the log2 fold-changes (FC) and the y-axis specifies the negative logarithm to the base 10 of the adjusted *p*-values. Gray vertical and horizontal dashed lines reflect the filtering criteria. Red and green dots represent genes expressed at significantly higher or lower levels, respectively. **(B)** Top 10 Gene ontology enrichment terms for up-regulated (top) and down-regulated (bottom) genes, respectively.

**Figure 5:**
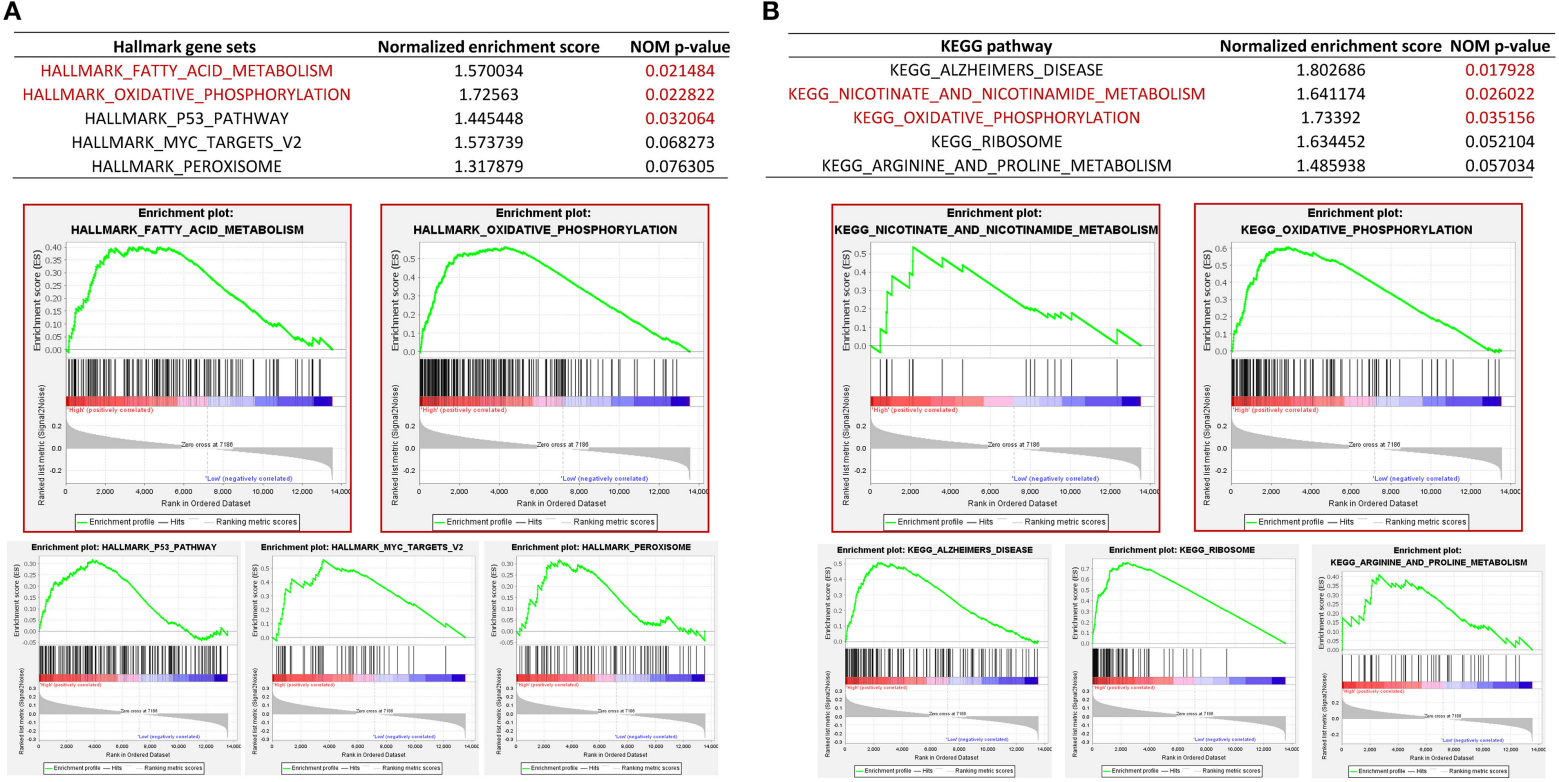
Gene set enrichment analysis between *PGRMC1* high- and low- expression samples. **(A)** GSEA comparing gene-expression signatures of TCGA HNSC tumors with the *PGRMC1* high- and low-expression by using hallmark gene sets. GSEA positive result table (top) showing the top five enrichment terms of the hallmark gene sets from MSigDB. **(B)** GSEA comparing gene-expression signatures of TCGA HNSC tumors with the *PGRMC1* high- and low-expression by using KEGG pathway gene sets. GSEA positive result table (top) showing top five enrichment terms of the KEGG pathway gene sets from MSigDB.

To gain insights into the molecular mechanisms underlying pro-tumorigenic action of *PGRMC1* transcripts in head and neck cancer cells, we first performed the differential gene expression analysis based on DESeq2 (Love et al., 2014) framework between *PGRMC1* high-expression samples (*N* = 125) and *PGRMC1* low-expression samples (*N* = 125) in HNSC dataset from TCGA database (Figure 4A). Our analysis identified a total of 194 genes as significantly up-regulated genes. More interesting, based on the gene ontology (GO) enrichment analysis by using the up-regulated genes, as shown in Figure 4B, almost all the enriched functional terms (including xenobiotic metabolic process and glutathione metabolic process) were metabolism-related. Considering the up-regulated metabolic activity could promote cell survival during nutrient limitation, oxygen-deficient environment, and other stresses, the results indicated that multiple genes involved in tumor metabolic activity pathway may be activated concordantly with *PGRMC1* over-expression, and make a contribution to the cancer cell survival and proliferation. Besides, various cell differentiation, skin development, and muscle contraction processes were enriched among *PGRMC1* low-expression HNSC samples (Figure 4B). We further performed gene set enrichment analysis (GSEA) using the MSigDB hallmark gene sets (Subramanian et al., 2005) and KEGG pathway gene sets, which revealed that a large number of gene sets were positively enriched in samples harboring *PGRMC1* high-expression compared with *PGRMC1*-low expression samples. Among the three significantly enriched hallmark gene sets, two groups of metabolism-related gene sets (Fatty Acid Metabolism: genes encoding proteins involved in the metabolism of fatty acids and Oxidative Phosphorylation: genes encoding proteins involved in oxidative phosphorylation) were highly positively enriched in patients harboring *PGRMC1* high-expression patients (Figure 5A). Similarly, among the three significantly enriched KEGG pathway gene sets, two groups of metabolism activity related pathways (nicotinate and nicotinamide metabolism pathway and oxidative phosphorylation pathway) were highly positively enriched in patients harboring *PGRMC1* high-expression patients (Figure 5B).

**Figure 6:**
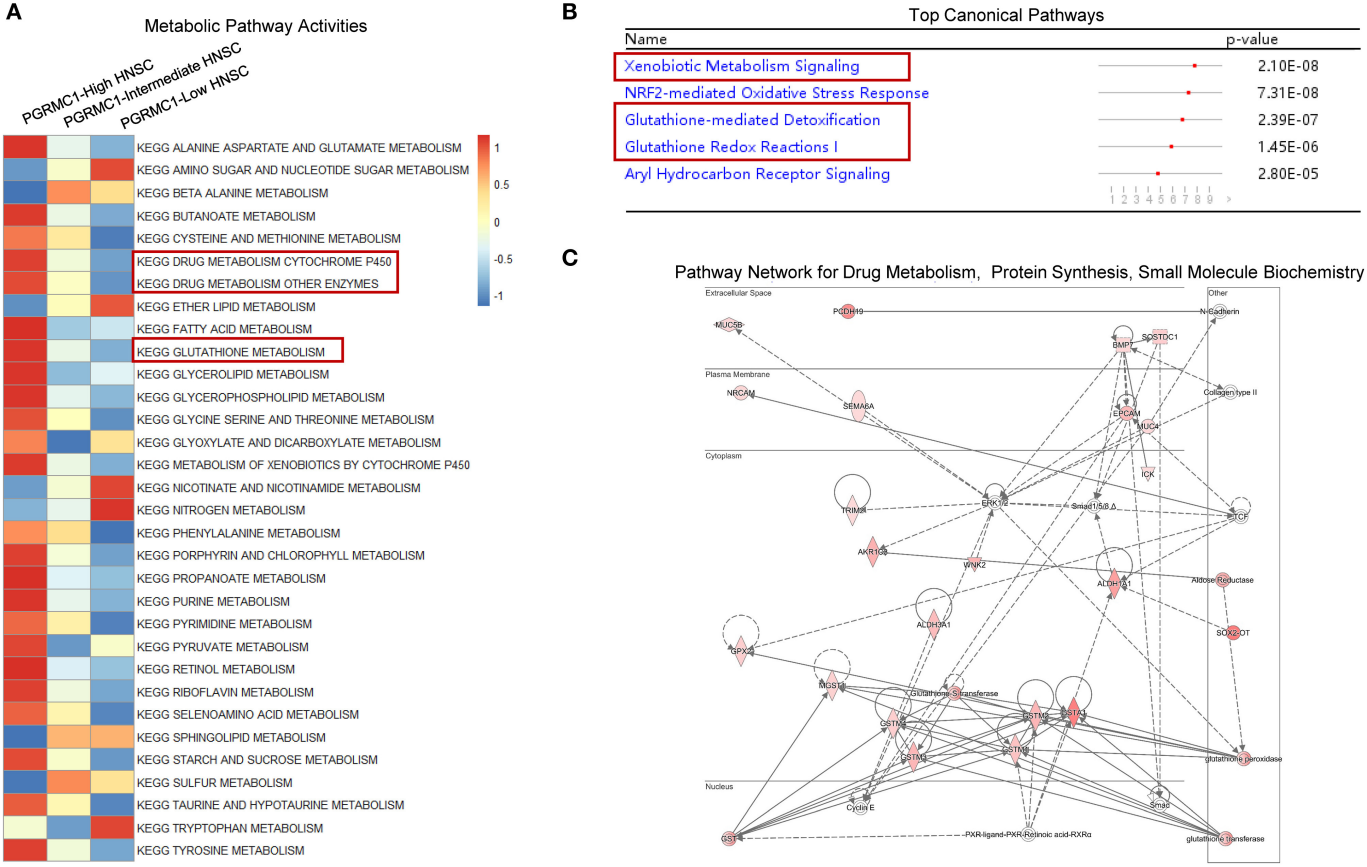
Metabolic pathway and network analysis. **(A)** Heat-map showed the mean score of a total of 32 metabolic pathways actively among the *PGRMC1* high-, intermediate-, and low-expression samples by using KEGG database. **(B)** Top canonical pathways determined by Ingenuity Pathway Analysis (IPA). **(C)** The highest-ranked signal pathway network revealed by Ingenuity Pathway Analysis (IPA). Proteins indicated in red were up-regulated in *PGRMC1*-high HNSC samples and the intensity of red means the fold change. The shapes are indicative of the molecular class (i.e., protein family). Lines connecting the molecules indicate molecular relationships. In detail, dashed lines indicate indirect interactions, and solid lines indicate direct interactions. The style of the arrows indicates specific molecular relationships and the directionality of the interaction (**A** acts on **B**).

Furthermore, to determine the general change of the metabolism pathway activity, a total of 32 metabolic pathways from KEGG database were selected to calculate the activity score based on the Gene-set Enrichment Analysis algorithm, the majority of 32 metabolic pathways were highly activated in *PGRMC1*-high HNSC samples (Figure 6A). Then we employed the Ingenuity Pathway Analysis (IPA) software to perform the canonical pathways enrichment and signal network analysis by using the up-regulated genes in *PGRMC1*-high HNSC samples. As shown in Figure 6B, among the top five enriched canonical pathways, three are metabolic-related (Xenobiotic metabolism signaling, Glutathione-mediated detoxification, Glutathione redox reactions I), which were consistent with GSEA pathway activity heatmap results. And the highest-ranked signal network was predominately associated with drug metabolism, protein synthesis, small molecule biochemistry (Figure 6C). In summary, these data demonstrate a strong positive correlation between *PGRMC1* expression and tumor metabolism activity, a key step in oncogenesis.

### Samples With High Expression of *PGRMC1* Exhibit a Selective Increase of PIK3CA Genomic Alterations

**Figure 7:**
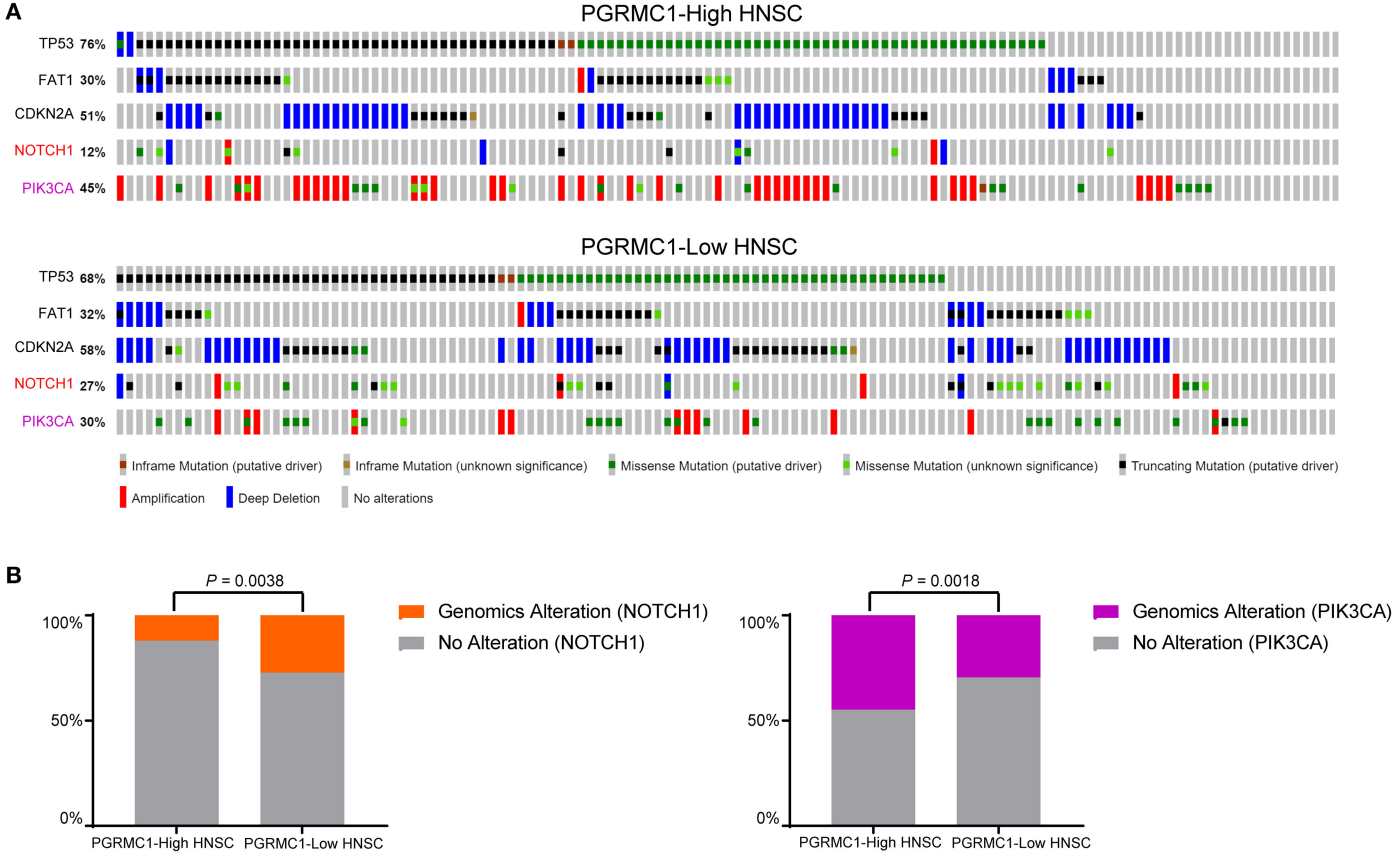
PIK3CA genomic alterations are selectively enriched in *PGRMC1*-high expression samples. **(A)** cBioPortal OncoPrint plot showing the distribution of TP53, FAT1, CDKN2A, NOTCH1, and PIK3CA genomic alterations rate in the TCGA HNSC dataset. **(B)** Bar graphs showing the percentage of TCGA HNSC samples with genomic alterations in NOTCH1 and PIK3CA by different *PGRMC1* expression groups.

To investigate whether *PGRMC1* over-expression cancers were enriched for some specific genomic alterations of conventional driver genes, we evaluated its association with the mutation profile of the five most high-frequency altered genes (TP53, FAT1, CDKN2A, NOTCH1, and PIK3CA) in HNSC. We employed the OncoPrint function of an online webserver called cBioPortal for Cancer Genomics tool to explore the genomic alterations rate of these five genes in the HNSC dataset (Figure 7A). Remarkably, as shown in Figure 7B, the genomic alterations rate of PIK3CA in the *PGRMC1* high-expression sub-group is 1.5-fold higher than the samples of low-expression sub-group, which reached nearly 45% of the total samples for HNSC cohort. In contrast, the tumor samples harboring *PGRMC1* high-expression have reduced the genomic alterations rate of NOTCH1 genes (Figure 7B). As many studies reported that HNSC with concurrent mutation of multiple PI3K pathway genes were advanced (stage IV), implicating concerted PI3K pathway aberrations in HNSC progression (Qiu et al., 2006; Lui et al., 2013), these data revealed the positive correlation between *PGRMC1* over-expression and PIK3CA genomic alterations, known for its association of cancer progression and metastasis, which provided further support for the oncogenic role of *PGRMC1* and the potential interaction with PIK3CA.

### Clinical Relevance of *PGRMC1* in HNSC Patients

**Figure 8:**
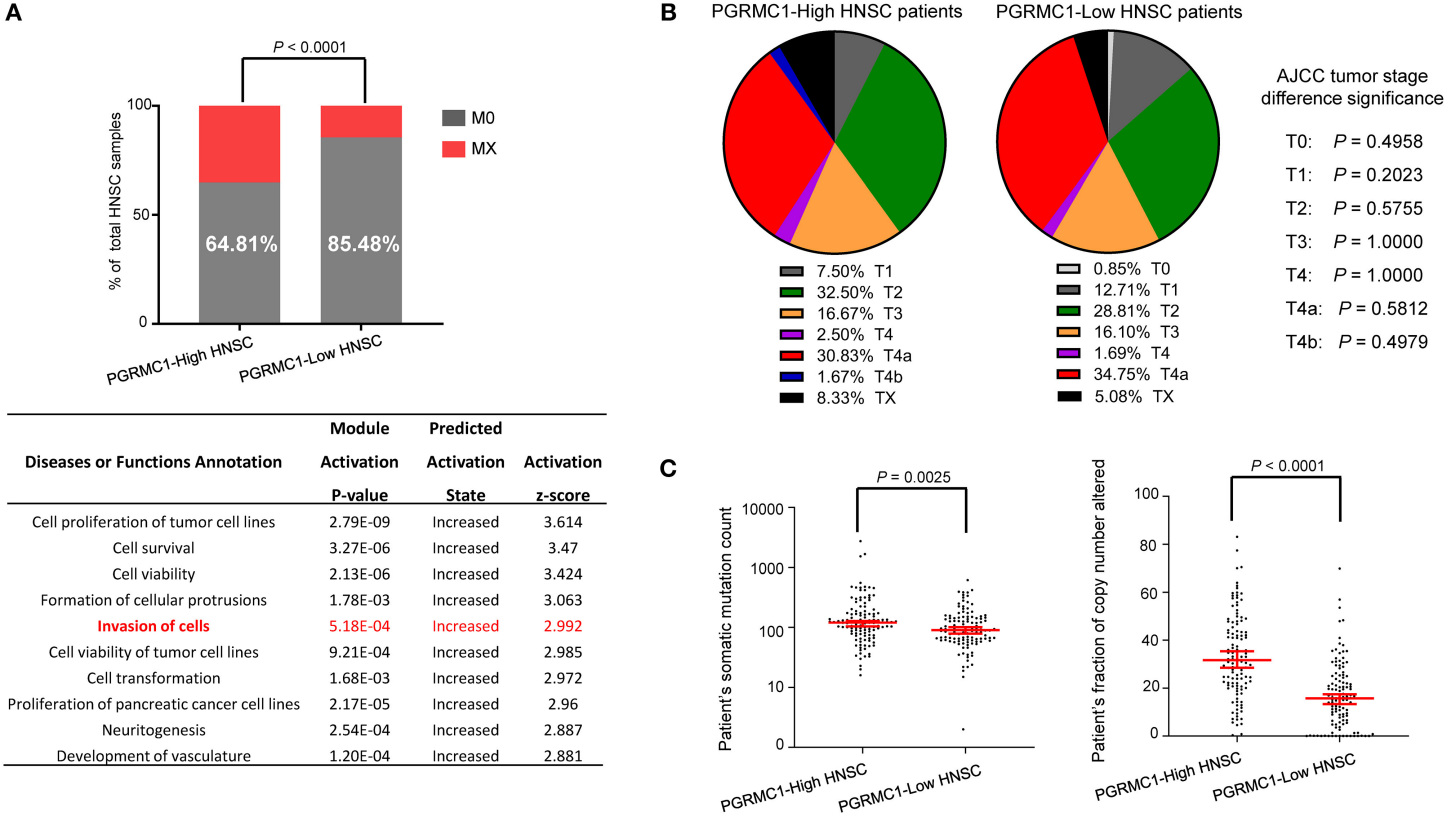
The difference in clinical characteristics between *PGRMC1* high- and low- expression samples. **(A)** Distribution of M0 and MX samples among different *PGRMC1* expression subtypes of HNSC from the TCGA patient cohort and functional modules activation analysis by IPA based on up-regulated genes in *PGRMC1*-high samples. **(B)** Distribution of tumor stages among different PGRMC1 expression subtypes. Statistical significance was determined by the Fisher's exact test. **(C)** The distribution plot of the patient's somatic mutation count (left) and the patient's fraction of copy number altered (right) among HNSC patients between *PGRMC1* high- and low- expression samples.

Head and neck cancer is a heterogeneous disease with diverse pathological and clinical features. We first investigated the distribution of metastasis stages among different *PGRMC1* expression groups. As shown in Figure 8A top, M0 (cancer has not spread to other parts of the body patients) samples were significantly enriched in groups with *PGRMC1* low-expression groups (*P* < 0.0001). Moreover, the functional modules activation analysis was performed by using IPA software with the up-regulated genes in *PGRMC1*-high HNSC samples as input, which revealed the module “Invasion of cells” was significantly activated (Figure 8A, bottom). However, there was no significant difference in tumor stage distribution (Figure 8B) and therapy-related clinical features (Supplementary Figure 2) between *PGRMC1* high-expression and low-expression groups. Remarkably, the tumors harboring *PGRMC1* high-expression showed the more abundant counts of somatic mutations and the fraction of genomic copy number alterations compared with *PGRMC1* low-expression tumors (Figure 8C).

## Discussion

Previous studies have shown *PGRMC1* is induced in many different types of cancers, including breast, thyroid, colon, ovary, and lung cancers (Hampton et al., 2015), and required for the key functions of tumor growth, cancer cell survival, and motility, particularly after chemotherapeutic drugs (Cahill et al., 2016). *PGRMC1* originally was identified by expression induction during carcinogenesis in liver cancer (Nie et al., 2006), and recently was observed its elevation with tumor stage in ovarian cancer and estrogen receptor-negative breast cancer (Neubauer et al., 2008). Despite the functional role of *PGRMC1* in aspects of tumor development, there is no sufficient evidence that *PGRMC1* expression is clinical application enough significant for various cancers. In this present study, we assessed the prognostic impact of aberrant *PGRMC1* expression in head and neck cancer and other cancers to identify patients who may benefit from anti-cancer therapy targeting *PGRMC1* and also investigated the oncogenic mechanism of *PGRMC1* over-expression in cancer cells. Considering the amplification is one of the major genetic mechanisms that could increase the gene expression of oncogenes (Savelyeva and Schwab, 2001; Lockwood et al., 2008; Zhang et al., 2019), it suggested that the copy number amplification of *PGRMC1* could serve as a potential genetic driver for its over-expression. Our data revealed, previously undescribed, the oncogenic and prognosis value of *PGRMC1* over-expression and copy number amplification in the head and neck cancer. It is convinced that the status of *PGRMC1* copy number amplification and over-expression will probably influence response to head and neck cancer treatment strategy.

*PGRMC1* promotes various phenotypes in the tumorigenesis, including tumor growth, invasion, metastasis, and apoptotic resistance. (Neubauer et al., 2013; Zhu et al., 2017). One intriguing possibility that *PGRMC1* facilitates participatory multiple oncogenic processes via some specific signaling pathways alterations. The hallmarks of cancer comprise many enhanced proliferation-related biological capabilities acquired during the multistep development of human tumors. It is usually initiated by the accumulated alterations of tumor metabolism signaling pathways, wherein the metabolic activation could provide the energetic and anabolic demands of proliferation, growth and even metastasis for cancer cells (Munoz-Pinedo et al., 2012; Martinez-Outschoorn et al., 2017).

Considering the cancer cells have the ability to rapidly proliferate by boosting conventional metabolic pathways for the synthesis of total biomass (proteins, lipids, and nucleic acids), and the maintenance of redox balance (DeBerardinis and Chandel, 2016; Renner et al., 2017), our present evidence of a strong correlation between up-regulation of *PGRMC1* expression and activation of multiple metabolism pathways reveal its essential function for the survival and growth of malignant cells. We believed our data reveal the potential mechanisms linking *PGRMC1* expression and altered metabolism pathway in tumorigenesis and metastasis of human head and neck cancers, which will contribute to the development of anti-cancer therapies that target cancer metabolism.

Furthermore, our data demonstrate that *PGRMC1* over-expressed in lung squamous cell carcinoma, kidney renal clear cell carcinoma, esophageal carcinoma and skin cutaneous melanoma, and plays an unfavorable prognosis role in head and neck cancer, breast cancer, acute myelocytic leukemia, and sarcoma. We believe all these findings uncover the potential of *PGRMC1* as an oncogene and therapeutic target for human cancer patients who over-express this gene.

## Data Availability Statement

The datasets used for the current study are available upon reasonable request from the corresponding author.

## Author Contributions

YZ designed the study, analyzed the data, and wrote the manuscript. XR helped perform the analysis with constructive discussions.

### Conflict of Interest

The authors declare that the research was conducted in the absence of any commercial or financial relationships that could be construed as a potential conflict of interest.

## Supplementary Material

Supplementary Figure 1 Expression distribution and Survival Significance of *PGRMC1*. **(A)** The distribution of *PGRMC1* expression value in the TCGA HSNC dataset. **(B)** Kaplan–Meier survival curve comparing the high (*N* = 249) and low (*N* = 249) expression value of *PGRMC1* (determined by the median value) for the TCGA HNSC patient cohort. **(C)** Kaplan–Meier survival curve comparing the samples with different copy number variation status of *PGRMC1* for the TCGA HNSC patient cohort. Statistical significance was determined by the log-rank test.

Supplementary Figure 2 The comparison of therapy-related clinical features between *PGRMC1* high- and low- expression samples. Distribution of three clinical therapy-related features among different *PGRMC1* expression subtypes of HNSC from the TCGA patient cohorts. Statistical significance was determined by the Fisher's exact test.

Supplementary Table 1 Cox regression analysis in TCGA HNSC patients.

## Abbreviations

TCGA: The Cancer Genome Atlas
HNSC: Head and Neck Squamous Cell carcinoma
HR: Hazard Ratio
OS: Overall Survival
GSEA: Gene-set Enrichment Analysis.
