# Supplementary figures and images for: Identification of PGRMC1 as a Candidate Oncogene for Head and Neck Cancers and Its Involvement in Metabolic Activities
Source: Front Bioeng Biotechnol. 2020 Jan 8;7:438. doi: 10.3389/fbioe.2019.00438 (PMC6960204; doi:10.3389/fbioe.2019.00438)

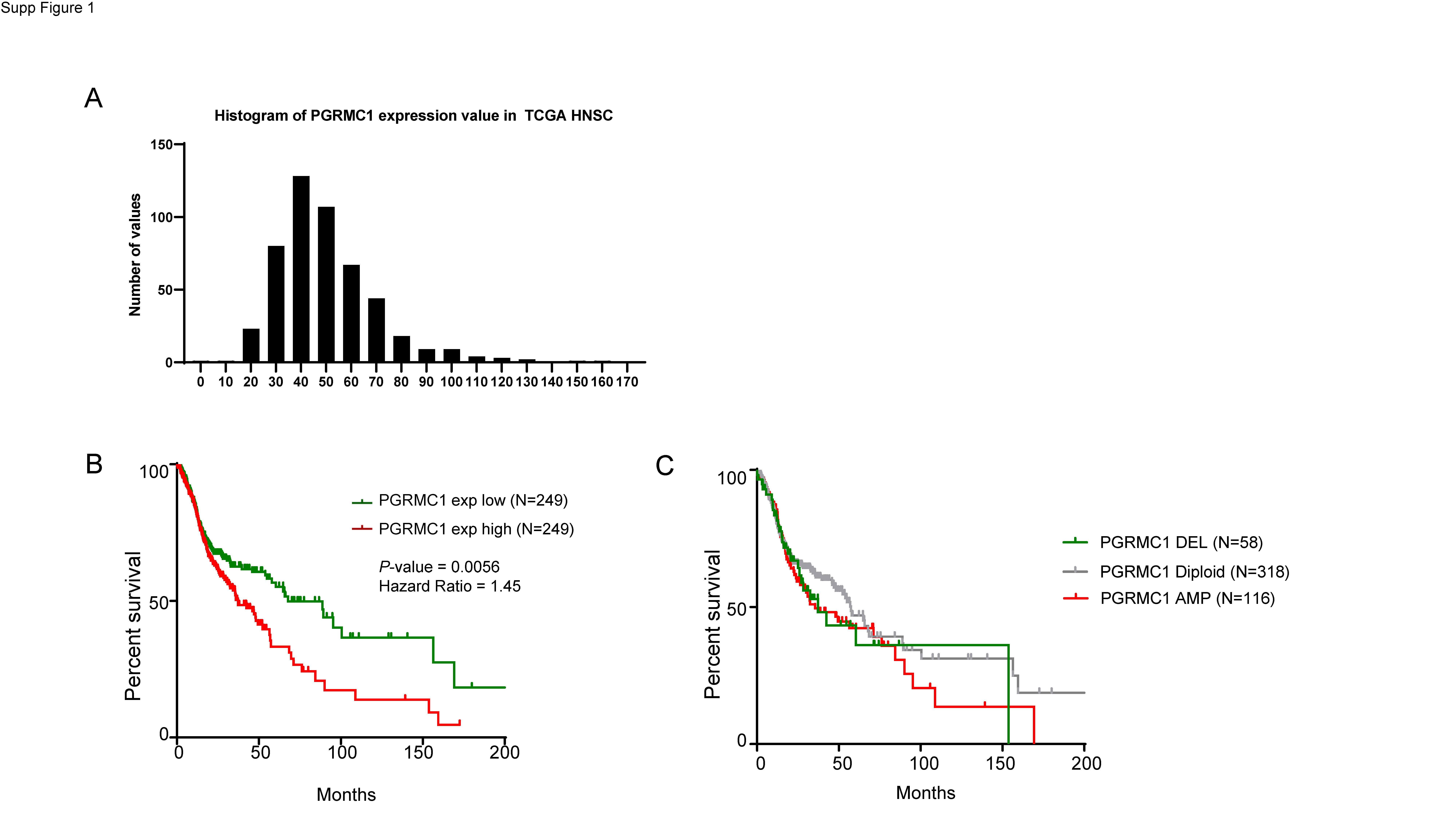

Supplement: Supplementary Figure 1 — Expression distribution and Survival Significance of PGRMC1. (A) The distribution of PGRMC1 expression value in the TCGA HSNC dataset. (B) Kaplan–Meier survival curve comparing the high (N = 249) and low (N = 249) expression value of PGRMC1 (determined by the median value) for the TCGA HNSC patient cohort. (C) Kaplan–Meier survival curve comparing the samples with different copy number variation status of PGRMC1 for the TCGA HNSC patient cohort. Statistical significance was determined by the log-rank test. [file Image_1.JPEG]

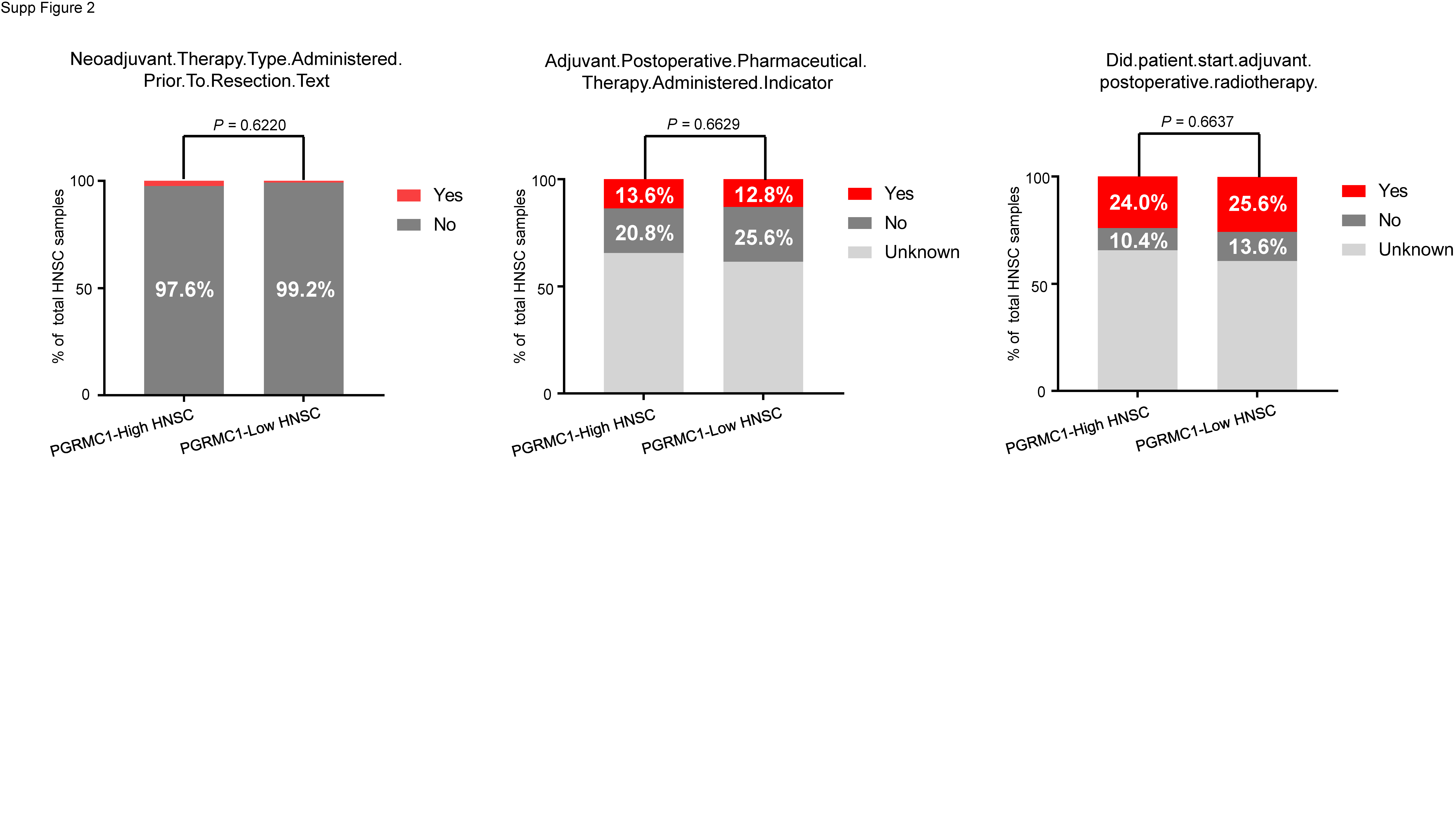

Supplement: Supplementary Figure 2 — The comparison of therapy-related clinical features between PGRMC1 high- and low- expression samples. Distribution of three clinical therapy-related features among different PGRMC1 expression subtypes of HNSC from the TCGA patient cohorts. Statistical significance was determined by the Fisher's exact test. [file Image_2.JPEG]
