# Supplementary material for: Identification of PGRMC1 as a Candidate Oncogene for Head and Neck Cancers and Its Involvement in Metabolic Activities
Source: Front Bioeng Biotechnol. 2020 Jan 8;7:438. doi: 10.3389/fbioe.2019.00438 (PMC6960204; doi:10.3389/fbioe.2019.00438)
Supplement: Supplementary Table 1 — Cox regression analysis in TCGA HNSC patients. [file Table_1.DOCX]

Supplementary Table 1 Cox regression analysis in TCGA HNSC patients

| **Characteristics** | | **Univariate** | |
| --- | --- | --- | --- |
|  |  | HR | *P*-value |
| **PGRMC1 expression value** | 1.001 | 0.773 |  |
| **PGRMC1 CNV Status**  Amp vs Low  Amp vs Diploid | 1.14  1.19 | 0.627  0.444 |  |
| **TP53 mutation (Mut vs WT)**  **Diagnosis Age** | 0.99  1.004 | 0.956  0.496 |  |
|  |  |  |  |
| **Patient Mutation Count**  **HPV status p16 (Pos vs Neg)**  **AJCC Metastasis Stage**  M1 vs M0  MX vs M0 | 1.0004  4.57  2.65  1.22 | 0.132  0.000265  0.333  0.298 |  |
| **AJCC Tumor Stage (vs T1)**  T2  T3  T4  T4a  T4b  TX | 0.66  0.51  2.44  0.61  1.11  0.42 | 0.093  0.0159  0.0179  0.0453  0.8861  0.0209 |  |
